# Correlation of Plasma 25(OH)D_3_ and Vitamin D Binding Protein Levels with COVID-19 Severity and Outcome in Hospitalized Patients

**DOI:** 10.3390/nu15081818

**Published:** 2023-04-10

**Authors:** Wajude Alabdullatif, Ahmad Almnaizel, Ali Alhijji, Aldanah Alshathri, Ahmed Albarrag, Iman Bindayel

**Affiliations:** 1 Department of Community Health Sciences, College of Applied Medical Sciences, King Saud University, Riyadh 11433, Saudi Arabia; wajude.alabdullatif@gmail.com (W.A.);; 2 Prince Naif for Health Research Center, King Saud University, Riyadh 11472, Saudi Arabia; 3 Division of Infectious Diseases, Department of Internal Medicine, College of Medicine, King Saud University, Riyadh 11461, Saudi Arabia; 4 Department of Pathology, College of Medicine, King Saud University, Riyadh 11461, Saudi Arabia

**Keywords:** COVID-19, 25(OH)D_3_, vitamin D binding protein, interleukin-6, interleukin-8, interleukin-10, tumor necrosis factor alpha

## Abstract

Background: The Coronavirus Disease-19 (COVID-19) caused by the Severe Acute Respiratory Syndrome Coronavirus-2 (SARS-CoV-2) has been declared a worldwide pandemic. The severity of COVID-19 varies greatly across infected individuals. Possible factors may include plasma levels of 25(OH)D and vitamin D binding protein (DBP), as both are involved in the host immune response. Other possible nutrition-related factors include malnutrition and/or obesity which disrupt the optimal host immune response to infections. Current literature shows inconsistent evidence about the association of plasma 25(OH)D_3_ and DBP on infection severity and clinical outcomes. Objectives: This study aimed to measure plasma 25(OH)D_3_ and DBP in hospitalized COVID-19 cases and assess their correlation with infection severity, inflammatory markers, and clinical outcome. Methods: 167 patients were included in this analytical cross-sectional study, of which 81 were critical and 86 were non-critical hospitalized COVID-19 patients. Plasma levels of 25(OH)D_3_, DBP, and the inflammatory cytokines, IL-6, IL-8, IL-10, and TNF-α were assessed using the Enzyme-linked Immunosorbent Assay (ELISA). Information regarding biochemical and anthropometrical indices, hospital length of stay (LoS), and illness outcome was obtained from the medical records. Results: Plasma 25(OH)D_3_ level was found to be significantly lower in critical compared to non-critical patients (Median = 8.38 (IQR = 2.33) vs. 9.83 (IQR = 3.03) nmol/L, respectively; *p* < 0.001), and it positively correlated with hospital LoS. However, plasma 25(OH)D_3_ did not correlate with mortality or any of the inflammatory markers. DBP on the other hand correlated positively with mortality (r_s_ = 0.188, *p* = 0.015) and hospital LoS (r_s_ = 0.233, *p* = 0.002). DBP was significantly higher in critical than non-critical patients (Median = 1262.18 (IQR = 463.66) vs. 1153.35 (IQR = 418.46) ng/mL, respectively; *p* < 0.001). Furthermore, IL-6 and IL-8 were significantly higher in critical than non-critical patients. However, no differences were found in IL-10, TNF-α, IL-10/TNF-α, TNF-α/IL-10, IL-6/IL-10, or CRP between groups. Conclusion: The current study found that critical COVID-19 patients had lower 25(OH)D_3_ than non-critical patients, yet, levels were found to be suboptimal in both groups. Further, critical patients had higher DBP levels as compared to non-critical patients. This finding may encourage future research to unravel the effects of this understudied protein that appears to have significant associations with inflammation, even though the precise mechanism is unknown.

## 1. Introduction

The Coronavirus Disease-19 (COVID-19) has spread worldwide and has been declared a pandemic by the World Health Organization (WHO). COVID-19 is caused by the Severe Acute Respiratory Syndrome Coronavirus-2 (SARS-CoV-2) [1]. This virus is an enveloped ribonucleic acid (RNA) coronavirus that binds to the Angiotensin Converting Enzyme 2 (ACE2) for host cell entry [1,2]. Upon infection, this virus can be either asymptomatic or cause a variable disease severity that can present as mild, moderate, or severe [3]. Most affected patients develop mild to moderate disease severity associated with fever, cough, headache, myalgia, and/or diarrhea [4,5]. With severe COVID-19 infection, patients may experience dyspnea as a result of hypoxemia [4]. Thereafter, respiratory failure develops which may associate with extrapulmonary diseases, such as gastrointestinal symptoms, cardiac and renal injury, cardiac arrhythmias, rhabdomyolysis, coagulopathy, shock, and eventually death [4,6].

Given the wide spectrum of severity, certain factors may be accountable for infection intensity, such as advanced age, male gender, obesity, history of smoking, hypertension, diabetes mellitus, coronary heart disease, chronic kidney disease (CKD), cerebrovascular disease, chronic obstructive pulmonary disease (COPD), malignancy, and chronic liver disease [3]. Yet, certain understudied factors that might contribute to the infection severity include 25(OH)D_3_ status and DBP level [7,8].

Recent reports have identified that SARS-CoV-2 may associate with long-term, extrapulmonary complications in addition to respiratory tract damage [9,10,11,12]. SARS-CoV-2 infection may impact male fertility by reducing semen quality and increasing levels of oxidative stress [9]. Moreover, SARS-CoV-2 can directly affect hepatocytes as a result of systemic inflammation, cytokine release syndrome, and hypoxia that are associated with the virus [10]. Other SARS-CoV-2-related complications include preeclampsia and neurological manifestations [11,12]. Essential processes required for placental development may be impaired as a result of SARS-CoV-2 infection. Indeed, the inflammatory response associated with SARS-CoV-2 infection is similar to that of preeclampsia, indicating a possible interaction between SARS-CoV-2 infection and the development of preeclampsia during pregnancy [11]. Furthermore, as SARS-CoV-2 causes systemic inflammation [6], acute and long-term neurological manifestations may arise [6,12]. Indeed, the inflammatory response associated with SARS-CoV-2 may damage the blood-brain barrier in addition to causing neuronal injury [12].

An upregulated immune response that involves elevated plasma levels of cytokines and chemokines is a risk factor for Cytokine Release Syndrome [6]. Elevated cytokine and chemokine levels have been observed in COVID-19 patients, increasing the risk for Cytokine Release Syndrome [6]. This syndrome is caused by an overactive immune response in which massive quantities of cytokines, including interleukin-1 (IL-1), interleukin-6 (IL-6), interleukin-18 (IL-18), interferon-gamma (IFN-γ), and tumor necrosis alpha (TNF-α) are expressed [13]. Serious consequences are associated with this syndrome and may include systemic inflammation, hyperferritinemia, hemodynamic instability, and multi-organ failure that necessitates intensive care unit (ICU) admission [14].

COVID-19 sufferers may also experience immunoparalysis [15]. Immunoparalysis is a state that is characterized by an altered innate immune response that persistently releases anti-inflammatory markers. Furthermore, this phenomenon has been recognized as a predictor of mortality in both adults and children [16]. Interleukin-10 (IL-10) and the ratios IL-10/lymphocyte count and IL-10/TNF-α have been recognized as markers for immunoparalysis [15].

Individuals suffering from COVID-19 may possibly benefit from having a sufficient plasma vitamin D level as this vitamin plays an essential role in immune function [17]. Indeed, adequate supplies are required to promote the growth and function of immune cells [17]. Vitamin D supports immunity by acting as an antioxidant, supporting the growth and activity of immune cells, and aiding in the production of antibodies [18]. The active form of vitamin D, 1,25(OH)_2_D modulates the immune responses by upregulating the expression of anti-inflammatory cytokines, downregulating pro-inflammatory cytokines expression, and regulating the production of antimicrobial proteins such as cathelicidin and β-defensin [19,20]. With respect to COVID-19, a systematic review showed that 25(OH)D level correlated negatively with disease incidence, severity, and mortality rate [21].

The vitamin D binding protein (DBP) is the primary transporter of vitamin D and its metabolites [22]. This protein is multifunctional and has other functions besides transporting vitamin D, such as having actin scavenging properties, an essential role in fatty acid transport, and immunomodulatory properties related to macrophage activation and neutrophil chemotaxis [23,24]. Actin is the most abundant cytoskeletal protein in all eukaryotic cells [25]. Great concentrations of actin are released into the extracellular fluid during acute respiratory distress syndrome (ARDS) because of extensive lung tissue damage and cellular death [26]. This can potentially induce lung inflammation that upregulates the inflammatory cascade [26]. The DBP was evaluated in one study on hospitalized COVID-19 patients and showed that DBP concentrations were significantly lower in patients who died [7].

Given the limited and inconsistent data regarding plasma 25(OH)D_3_ and DBP levels in hospitalized COVID-19 patients, the primary aim of this study was to measure 25(OH)D_3_ and DBP plasma levels in critical and non-critical hospitalized COVID-19 patients. The secondary aim was to correlate 25(OH)D_3_ and DBP with infection severity, particularly inflammatory markers, hospital length of stay (LoS), and outcome.

## 2. Materials and Methods

### 2.1. Study Design and Subjects

This analytical cross-sectional study was conducted on hospitalized COVID-19 patients who were admitted to King Saud University Medical City (KSUMC) from January to June 2021. Patients who were admitted to the COVID-19 ICU and COVID-19 wards were screened for eligibility. Eligible patients were aged 18 years and above, had a positive result from the COVID-19 PCR test and were hospitalized for COVID-19. Patients were excluded if they were vaccinated against COVID-19, were hospitalized for reasons other than COVID-19, were on chemotherapy, suffered from malabsorptive problems, were on dialysis or were pregnant or lactating.

The sample size was calculated based on detecting a significant difference in the primary outcome, 25(OH)D_3_, between groups. 25(OH)D level reported by Carpagnano et al. [8] was used for the calculation, with a two-sided significance level of 5% and a precision/absolute error of 6 nmol/L. The total sample size was equal to 180 participants, with 90 participants per group.

#### Criteria for Determining Disease Severity

Patients were deemed critical and admitted to the ICU based on the KSUMC general protocol for ICU admission. The protocol included four priority categories that were based on the possible benefits to the patient. The first priority included being critically ill, unstable, and in need of life-saving, intensive treatment such as invasive mechanical ventilation, need for vasoactive agents, and/or aggressive volume resuscitation.

KSUMC’s protocol included objective measures to assess patients’ eligibility for ICU admission. They were considered eligible and transferred to the ICU if they had one or more of the following: pulse of less than 40 or greater than 150 beats/min; systolic arterial pressure less than 80 mm Hg or 20 mm Hg below the patient’s usual pressure; mean arterial pressure less than 60 mm Hg; diastolic arterial pressure greater than 120 mm Hg; respiratory rate greater than 35 or less than 10 breaths/min; serum sodium levels below 110 or greater than 170 mEq/L; serum potassium less than 2 or greater than 7 mEq/L; PaO_2_ less than 50 mm Hg; PaCO_2_ less than 25 or greater than 50 mm Hg; pH less than 7.1 or greater than 7.7; serum glucose greater than 44 mmol/L; serum calcium greater than 3.75 mmol/L; blood urea nitrogen (BUN) greater than 35 mmol/L; creatinine greater than 88 mmol/L; urine output less than 20 mL/h; unstable hemoglobin (Hgb) that was equal to or less than 7 gm/dL (with the exception of chronic anemia) and/or greater than a 2 gm decrease within 24 h; toxic levels of drugs or other substances in a hemodynamically or neurologically unstable patient; cerebral vascular hemorrhage; ruptured viscera, bladder, or liver with hemodynamic instability; dissecting aortic aneurism; airway obstruction; cyanosis; coma and seizures.

Patients were deemed non-critical if they had a mild to moderate or severe disease severity that required hospital admission without critical care.

### 2.2. Study Outcomes

The primary outcome of the study was plasma 25(OH)D_3_ level between groups. Other outcomes consisted of plasma levels of DBP and the proinflammatory markers, IL-6, IL-8, TNF-α, and CRP, anti-inflammatory marker IL-10, and their ratios, IL-10/TNF-α, TNF-α/IL-10, and IL-6/IL-10 between groups. Additionally, secondary outcomes also consisted of hospital LoS and mortality rates across groups. Furthermore, the correlation between variables collected was evaluated to generate associations between the relationship of 25(OH)D_3_ and DBP with disease severity and outcome.

### 2.3. Data Collection

Information pertaining to age, gender, ethnicity, medical history, routine laboratory tests, vital signs (blood pressure, heart rate, temperature, O_2_ saturation), and hospital LoS were collected from the medical files of patients.

#### Laboratory Tests

Blood samples were collected to measure plasma levels of 25(OH)D_3_, DBP, IL-6, IL-8, IL-10, and TNF-α. Samples were mostly obtained within 24–72 h after admission to the ICU or ward; 10 mL of whole blood was extracted from each patient by the venipuncture method or from a venous line. Ethylenediaminetetraacetic acid (EDTA) blood was centrifuged (2000× *g*, 15 min) immediately after sample collection to prevent any degradation or absorption. Plasma samples were then frozen at −80 °C for analysis at the end of data collection.

Samples were analyzed at the end of the study by using the human ELISAs for 25(OH)D_3_, DBP, IL-6, IL-8, IL-10, and TNF-α. White blood cell (WBC) count, HGB, hematocrit (HCT), albumin, alanine transaminase (ALT), aspartate aminotransferase (AST), creatinine, bilirubin total, sodium, potassium, random blood glucose (RBG), troponin, CRP, D-dimer, activated partial thromboplastin time (APTT), international normalized ratio (INR), and prothrombin time (PT) were obtained from patients’ routine hospital laboratory records upon admission to the ICU or ward.

25(OH)D_3_ (NBP2-66361, Novus Bio, Centennial, CO, USA), DBP (DVDBP0, R&D Systems, Inc., Minneapolis, MN, USA), IL-6 (D6050, R&D Systems, Inc., Minneapolis, MN, USA), IL-8 (D8000C, R&D Systems, Inc., Minneapolis, MN, USA), IL-10 (MBS764410, My BioSource, San Diego, CA, USA), and TNF-α (MBS2502004, My BioSource, San Diego, CA, USA) were analyzed by the ELISA technique. The kits employ competitive-ELISA as the method for determination. All analyses were performed according to the manufacturer’s instructions. To avoid interindividual variability, all samples were analyzed by the same researcher.

### 2.4. Statistical Analysis

Statistical analysis was performed using the Statistical Package for Social Sciences (SPSS) version 28.0 (IBM-SPSS, Armonk, NY, USA). Normality testing was performed for all continuous variables. Log-10 transformation was performed for all continuous non-parametric variables to achieve normality and allow for parametric tests to be conducted. After log transformation, variables were checked once more for normality.

Results were expressed as a number and percentage for categorical variables and mean and standard deviation or median and interquartile range for continuous variables. The correlation was conducted using the pearson correlation test for parametric data; however, the spearman correlation test was used for non-parametric and categorical variables. A chi-squared test was used to determine the difference in proportions for categorical variables. The difference in the mean between groups was assessed using the independent samples t-test for normally distributed data. In contrast, the mann–whitney U–test was used for non-parametric data. A *p*-value of <0.05 was considered statistically significant.

## 3. Results

### 3.1. Demographic and Clinical Characteristics of the Sample

This study included a total of 167 COVID-19-positive patients. Of the 167, 86 were non-critical and 81 were critical. The critical group was significantly older than the non-critical group (62.6 ± 15.9 vs. 57.6 ± 13.9 years, respectively; *p* = 0.031). However, no differences were found in gender distribution between the two groups (Table 1).

At least one comorbidity was present in 89.8% of patients. The most frequent comorbidity was diabetes mellitus, followed by hypertension, then obesity (54.5%, 51.5%, and 48.5%, respectively). No significant differences were found in rates of comorbidities between groups, except for cardiovascular disease, which was significantly higher in the critical versus non-critical group (23.5% vs. 7%, respectively; *p* = 0.003) (Table 1).

The biochemical indices of the two groups are shown in Table 2. The critical group had significantly higher WBC and platelet counts and lower albumin compared to the non-critical group (Table 2). Moreover, D-dimer was significantly higher in the critical compared to the non-critical group (Median = 1.29 (IQR = 1) vs. 0.95 (IQR = 1) mcg/mL, respectively; *p* < 0.001).

### 3.2. 25(OH)D_3_, DBP, and Inflammatory Markers

25(OH)D_3_ level was significantly higher in the non-critical versus critical group (Median = 9.83 (IQR = 3.03) vs. 8.38 (IQR = 2.33) nmol/L, respectively; *p* < 0.001, Table 3). Moreover, DBP level was significantly greater in the critical compared to the non-critical group (Median = 1262.18 (IQR = 463.66) vs. 1153.35 (IQR = 418.46) ng/mL, respectively; *p* = 0.001, Table 3).

Plasma levels of the inflammatory markers, IL-6 and IL-8 were significantly higher in the critical compared to the non-critical group (Table 3). No differences were found with IL-10, TNF-α, IL-10/TNF-α, TNF-α/IL-10, or IL-6/IL-10 (Table 3).

### 3.3. Clinical Outcomes of Cases

Critical cases had a mean ICU stay of 11.85 ± 7.89 days. Additionally, they had a significantly longer mean hospital LoS compared to non-critical patients (Table 4). In addition, their mortality rate was significantly higher than non-critical patients (40% vs. 1.2%, respectively; *p* < 0.001). Moreover, critical patients who died had a mean hospital LoS stay of 28 ± 22.5 days before death (Table 4).

### 3.4. Correlation Analysis

There was a significant and negative association between 25(OH)D_3_ plasma level and hospital LoS (r_s_ = −0.342, *p* = 0.002). However, no associations were detected with age, DBP, IL-6, IL-8, IL-10, TNF-α, IL-10/TNF-α, TNF-α/IL-10, IL-6/IL-10, CRP, mortality, age, or BMI.

Plasma DBP correlated positively with hospital LoS (r_s_ = 0.233, *p* = 0.002), age (r_s_ = 0.155, *p* = 0.046), and mortality (r_s_ = 0.188, *p* = 0.015). Yet, no correlation was detected with IL-6, IL-8, IL-10, TNF-α, IL-10/TNF-α, TNF-α/IL-10, IL-6/IL-10, CRP, or BMI.

## 4. Discussion

The present study found significantly lower 25(OH)D_3_ and higher DBP levels in critical compared to non-critical patients. Although, the difference in 25(OH)D_3_ between groups was small. Such findings might be attributed to illness severity [27,28].

Research that has assessed total 25(OH)D levels in hospitalized COVID-19 patients in Saudi Arabia has reported the prevalence of insufficiency/deficiency to be between 62.6 and 73% [29,30,31], while in the United Arab Emirates, it was reported to be 66.6% [32].

In the present study, the level of 25(OH)D_3_ was considered to be suboptimal in both groups, as the median level of the sample was equal to 9.08 nmol/L. This might be attributed, in part, to the infection as vitamin D may serve as a negative acute phase reactant during acute and chronic inflammatory conditions [28]. Still, other understudied factors such as inadequate sun exposure, skin type, age, weight, inadequate dietary intake, or malabsorption may influence 25(OH)D status independent of the infection [33,34].

Merzon et al. (2020) found the risk for hospitalization from COVID-19 to be greater in individuals with lower 25(OH)D levels [35]. With respect to 25(OH)D_3_ levels, symptomatic COVID-19-positive patients had significantly lower 25(OH)D_3_ levels than COVID-19-negative controls [36].

The results of the present study showed that 25(OH)D_3_ plasma level was negatively associated with hospital LoS in COVID-19 patients; however, it was not associated with mortality. Similarly, Nguyen and colleagues (2022) have evaluated 25(OH)D_3_ in hospitalized COVID-19 patients and identified that low levels of 25(OH)D_3_ were significantly associated with an increased hospital LoS [37]. On the other hand, Orchard et al. [38] reported no significant differences in hospital LoS and mortality between critically ill COVID-19 patients with 25(OH)D levels below or above 50 nmol/L.

Five inflammatory markers were evaluated in the present study, IL-6, IL-8, IL-10, TNF-α, and CRP in hospitalized COVID-19 patients. However, the results yielded no associations with 25(OH)D_3_ levels. Other studies have reported similar findings and have not detected any associations between 25(OH)D status and inflammatory markers [29,38,39]. Contrarily, Gallelli et al. [36] found a positive correlation between 25(OH)D_3_ and IL-6 levels in symptomatic COVID-19 patients who had a 25(OH)D_3_ level below 2.5 nmol/L. Positive associations between IL-6 and 25(OH)D_3_ could be the result of limitations in the study that included performing the analysis on a sub-sample of only six patients. Additionally, the IL-6 level was shown to be low in those cases (3.16 pg/mL) [36].

The DBP is a multifunctional protein mainly produced by hepatocytes and it is the main transporter of all vitamin D metabolites including 25(OH)D_3_ [22,40]. Plasma DBP level may remain stable or is marginally enhanced during the acute phase of inflammation, as opposed to albumin, which is reduced during periods of physiological stress [27]. Contrarily, other reports indicate that inflammation may be associated with lower DBP levels [41,42]. With respect to the current study, plasma DBP level was significantly higher in critical compared to non-critical patients, which contrasts with the findings of Subramanian et al. [7]. Moreover, a higher DBP level was associated with a longer hospital LoS. As previously described, the severity of the illness could be a contributing factor to a higher plasma DBP level.

Another factor that could explain higher DBP in critical patients was possible acute liver damage. DBP levels may increase as a result of acute liver failure [43]. ALT level in critical patients was greater than in non-critical patients. Greater ALT level may be an indicator of liver injury [44], which may explain why a higher DBP plasma level was found in critical rather than non-critical patients.

Neutrophils are the primary white blood cells that play a key role in host defense. The recruitment of neutrophils to tissue is mediated through various chemoattractants. In vivo and in vitro work have both shown that DBP upregulates the chemotactic activity of neutrophil chemoattractants [27]. During normal conditions, around 1–2% of circulating DBP is vitamin D bound. Given that the DBP plays an important role in the actin scavenging system [27], we hypothesize that during periods of inflammation, great amounts of actin can bind to DBP since only 1–2% of vitamin D is bound to it. Contrarily, research suggests that during periods of increased stress, the body produces large amounts of actin which bind to DBP thereby reducing its concentrations as a result of extensive consumption [45]. Yet, following periods of trauma and increased stress, DBP levels may in fact increase as a result of increased concentrations of cytokines and glucocorticoids. The increase in DBP is preceded by an initial reduction as a result of actin scavenging [40].

It is not fully understood why critical COVID-19 patients in our sample had significantly higher DBP than non-critical patients or why a positive association between DBP level and hospital LoS was detected. Although differences in plasma DBP between groups could be related to COVID-19 severity, the mechanism is not fully understood. These variations could possibly be due to possible liver injury, interindividual differences among patients, and/or gene polymorphisms related to the DBP [43,46]. The DBP gene is considered to be the most polymorphic protein, which can affect its functions [46]. Therefore, patients in the sample may have had different DBP polymorphisms which could have influenced the outcome of the results.

This study is among the few that have assessed 25(OH)D_3_ and DBP in hospitalized COVID-19 patients and related them to the severity of illness. Efforts were undertaken to reduce the risks of confounding factors and included excluding patients who had received the COVID-19 vaccine were on dialysis or chemotherapy, had malabsorption problems, and were pregnant or lactating. Given the key strengths mentioned, this study has limitations that are recognized. For instance, the study was observational in nature and was conducted at a single center, therefore, limiting its generalizability. Nonetheless, the sample may be heterogenous since it included ICU patients. Additionally, heterogeneity may be caused by the fact that the study included all hospitalized COVID-19 patients regardless of their preexisting medical condition, except for those with one of the previously mentioned exclusion criteria.

## 5. Conclusions

In conclusion, this research found that critical patients had lower 25(OH)D_3_ levels than non-critical patients, although most of the hospitalized COVID-19 patients had suboptimal levels. Though significant, the difference in 25(OH)D_3_ between groups was very small. Nonetheless, our findings showed that DBP was higher in critical than non-critical patients, in addition to being directly associated with a longer hospital LoS. While the exact mechanism may not be fully understood, it may prove fruitful for future research to unravel the effects of this understudied protein that appears to be substantially associated with inflammation. Future research should focus on studying the exact mechanism of DBP and inflammation during critical illness. Additionally, these studies should measure actin and evaluate its effect on plasma DBP levels. Additionally, larger studies should be undertaken to determine normal DBP levels in both healthy and ill individuals. Finally, more research should be encouraged that groups patients based on 25(OH)D level to determine whether there is an observable association on disease outcomes.

## Figures and Tables

**Table 1:** Baseline characteristics of study groups.

Characteristics	Total (n = 167)	Non-Critical (n = 86)	Critical (n = 81)	*p* Value
Age, years—Mean ± SD	59.54 ± 14.84	57.58 ± 13.90	62.62 ± 15.9	0.031
Age ≥ 65 years	63 (37.7)	25 (29.1)	38 (46.9)	0.017
Females	84 (50.3)	46 (53.5)	38 (46.9)	0.396
Males	83 (49.7)	40 (46.5)	43 (53.1)	
Weight, kg—Mean ± SD	82.66 ± 19.6	82.11 ± 18.51	83.36 ± 20.5	0.678
BMI, kg/m^2^—Mean ± SD	30.87 ± 7.17	30.46 ± 0.67	31.31 ± 8.08	0.447
Vital signs upon admission—Mean ± SD				
Systolic BP, mm Hg	124.59 ± 16.4	125.14.45	123.65 ± 18.5	0.431
Diastolic BP, mm Hg	68.11 ± 9.43	69.26 ± 9.20	66.75 ± 9.38	0.084
Temperature, °C	36.99 ± 0.52	37.04 ± 0.57	36.94 ± 0.45	0.218
Respiratory rate, BPM	25.28 ± 6.23	21.54 ± 2.73	29.44 ± 6.46	<0.001
O_2_ saturation, %	93.29 ± 4.35	95.05 ± 1.93	91.21 ± 2.56	<0.001
Comorbidities				
Any comorbidity	150 (89.8)	77 (89.5)	73 (90.1)	0.900
Diabetes mellitus	91 (54.5)	43 (50.0)	48 (59.3)	0.230
Hypertension	86 (51.5)	44 (51.2)	42 (51.9)	0.929
Obesity	81 (48.5)	46 (53.5)	35 (43.2)	0.184
Chronic kidney disease	8 (4.8)	4 (4.7)	4 (4.9)	0.931
Cardiovascular disease	25 (15.0)	6 (7.0)	19 (23.5)	0.003
Previous cardiovascular disease	17 (10.2)	7 (8.1)	10 (12.3)	0.369
Respiratory illnesses	32 (19.2)	14 (16.3)	18 (22.2)	0.329
Liver diseases	6 (3.6)	2 (2.3)	4 (4.9)	0.365
Nervous system diseases	8 (4.8)	3 (3.5)	5 (6.2)	0.417

Note: Groups were compared using the chi-square test, except for age, weight, BMI, and vital signs which were analyzed using the independent samples *t*-test. Variables are presented as N (%), unless otherwise stated. A *p*-value < 0.05 is considered significant. BMI, body mass index; BP, blood pressure; BPM, beats per minute; SD, standard deviation.

**Table 2:** Biochemical indices of the critical and non-critical groups.

Variable	Total (n = 167)	Non-Critical (n = 86)	Critical (n = 81)	*p* Value
WBC count, x 10^9^/L	8.11 ± 3.93	6.04 ± 2.50	9.39 ± 4.32	<0.001
Hgb, g/L	130.80 ± 20.61	131.83 ± 16.37	128.88 ± 24.93	0.366
HCT, %	39.35 ± 4.54	39.04 ± 4.76	38.96 ± 5.93	0.921
Platelets, x 10^9^/L	246.73 ± 104.29	217.39 ± 83.55	265.43 ± 113.76	0.002
ALT ^1^, unit/L	50.86 ± 33.6	49.19 ± 30.22	52.61 ± 36.93	0.720
AST ^1^, unit/L	65.12 ± 42.12	57.85 ± 35.48	72.76 ± 47.13	0.011
Bilirubin total ^1^, umol/L	8.44 ± 4.08	8.19 ± 3.69	8.71 ± 4.45	0.204
Albumin, g/L	27.94 ± 4.33	30.07 ± 4.31	26.60 ± 3.86	<0.001
Creatinine ^1^, mcmol/L	90 ± 46.94	84.38 ± 40.57	97.53 ± 52.34	0.033
Sodium, mmol/L	136.91 ± 4.04	135.86 ±4.16	137.32 ± 4.69	0.035
Potassium, mmol/L	4.38 ± 0.68	4.22 ± 0.62	4.66 ± 0.88	<0.001
RBG, mmol/L	9.57 ± 5.18	8.74 ± 4.77	10.18 ± 5.32	0.067
D-dimer, mcg/mL—Median (IQR)	1.11 (1)	0.95 (1)	1.29 (1)	<0.001
APTT, seconds	39.54 ± 9 12.09	39.09 ± 9.94	40.89 ± 11.83	0.309
INR, seconds	1.08 ± 9 0.74	0.97 ± 0.18	1.12 ± 0.71	0.073
PT, seconds	15.02 ± 9.35	13.61 ± 2.44	15.56 ± 9.1	0.077

Note: Groups were compared using the independent samples *t*-test, except for D-dimer which was analyzed using the mann–whitney U–test. Variables are presented as mean ± SD, unless otherwise stated. A *p*-value < 0.05 is considered significant. ^1^ Variables are log transformed to achieve normality. ALT, alanine transaminase; AST, aspartate aminotransferase; HCT, hematocrit; HGB, hemoglobin; INR, international normalization ratio; PT, prothrombin time; RBG, random blood glucose; WBC, white blood cell.

**Table 3:** Differences in 25(OH)D_3_, DBP, and inflammatory markers between groups.

Variable	Total (n = 167)	Non-Critical (n = 86)	Critical (n = 81)	*p* Value
25(OH)D_3_, nmol/L	9.08 (2.72)	9.83 (3.03)	8.38 (2.33)	<0.001
DBP, ng/mL	1204.84 (427.52)	1153.35 (418.46)	1262.18 (463.66)	0.001
IL-6, pg/mL	24.63 (4.3)	23.93 (2.75)	26.80 (7.57)	<0.001
IL-8, pg/mL	71.01 (153.59)	32.47 (92.15)	124.55 (245.89)	<0.001
IL-10, pg/mL	266.23 (456.53)	206.76 (458.82)	248.76 (479.08)	0.674
TNF-α, pg/mL	131.17 (227.25)	124.92 (171.26)	151.88 (418.06)	0.125
IL-10/TNF-α	1.73 (2.96)	1.85 (2.85)	1.53 (3.05)	0.315
IL-6/IL-10	0.1334 (0.23)	0.12 (0.21)	0.134 (0.24)	0.353
TNF-α/IL-10	0.61 (1.16)	0.78 (1.37)	0.76 (2.06)	0.220
CRP, mg/L	103.5 (110.6)	88.95 (112.5)	113.0 (120.13)	0.057

Note: Data were analyzed using the mann–whitney U–test. Variables are presented as median (IQR). A *p*-value < 0.05 is considered significant. CRP, C-reactive protein; DBP, vitamin D binding protein; IL-6, interleukin 6; IL-8, interleukin 8; IL-10, interleukin 10; TNF-α, tumor necrosis factor alpha; 25(OH)D_3_, 25-hydroxyvitamin D3.

**Table 4:** Differences in clinical outcomes between groups.

Outcome	Total (n = 167)	Non-Critical (n = 86)	Critical (n = 81)	*p* Value
Hospital LoS, days	12.57 ± 12.8	5.95 ± 2.76	24.29 ± 15.1	<0.001
ICU LoS, days	-	-	11.85 ± 7.89	
Mortality—N (%)	34 (20.4%)	1 (1.2%)	33 (40.7%)	<0.001
Length of hospital stay until mortality, days	-	-	28.03 ± 22.5	

Note: The chi-square test was used to compare mortality between groups and the independent samples *t*-test was used for LoS. Variables are presented as mean ± SD, unless otherwise stated. A *p*-value < 0.05 is considered significant. ICU, intensive care unit; LoS, length of stay.

## Data Availability

The data presented in this study are available only upon request from the corresponding author.
